# Matrilineal Behavioral and Physiological Changes following the Removal of a Non-Alpha Matriarch in Rhesus Macaques (*Macaca mulatta*)

**DOI:** 10.1371/journal.pone.0157108

**Published:** 2016-06-08

**Authors:** Lauren J. Wooddell, Stefano S. K. Kaburu, Kendra L. Rosenberg, Jerrold S. Meyer, Stephen J. Suomi, Amanda M. Dettmer

**Affiliations:** 1 Eunice Kennedy Shriver National Institute for Child Health and Human Development, National Institutes of Health, Poolesville, Maryland, United States of America; 2 Department of Population Health and Reproduction, School of Veterinary Medicine, University of California Davis, Davis, California, United States of America; 3 Department of Psychological and Brain Sciences, University of Massachusetts Amherst, Amherst, Massachusetts, United States of America; Tulane University, UNITED STATES

## Abstract

In many species, the loss of alpha matriarchs is associated with a number of negative outcomes such as troop fission, eviction, wounding, and reduced vitality. However, whether the dramatic consequences of their loss are due to their role as an old experienced figure or to their alpha status remains unclear. In a retrospective study, we tested that in a semi-free ranging colony of rhesus macaques (*Macaca mulatta*), the removal of a non-alpha matriarch, who had a large set of kin, led to changes in behavior and physiological stress within her matriline. Following her removal, her matriline increased in aggression, vigilance, and social grooming. Additionally, hierarchical stability, measured by levels of rank changes, decreased within her matriline, and levels of intense aggression by high-ranking animals were more frequent, as well as matrilineal wounding. Although ordinal rank was positively associated with higher chronic hair cortisol concentrations (HCCs) in the months before the matriarch’s removal, following her removal, only those who experienced large increases in rank within her matriline displayed higher HCCs. Changes in matrilineal stability, aggression, behavior, and HCCs within the other two matrilines in the troop were not evident, although caution is needed due to the small sample sizes. We conclude that the removal of the non-alpha matriarch led to matrilineal instability, characterized by higher levels of aggression and subsequent vigilance, rank changes, physiological stress, and grooming. We suggest that non-alpha matriarchs with a large number of kin and social support can be integral to the stability of matrilines.

## Introduction

Among long-living animals, old females can play a crucial role for the survival of their family or social group. This support can come indirectly if these females use the experience they have accumulated over the years to lead the group and protect it from external threats [1–3] or directly by providing care to the future generations [4] or, at least in some cases, by controlling intra-group conflicts [5]. For example, in African elephants (*Loxodonta africana*), family units are commonly led by the oldest female, who exhibits the necessary social and ecological knowledge to coordinate her family and better recognize dangers [1, 6–8]. Accordingly, older female elephants were found to be better at discriminating between familiar and stranger groups [6], and at displaying more appropriate responses to predators [1] than young females, which can explain why old females tend to have larger families with more calves [6]. Similarly, among orcas (*Orcinus orca*), menopausal old females use their ecological knowledge to guide their families towards feeding sources, especially during periods of food scarcity [2], a trait that likely increases their offspring’s survival [9]. In chimpanzees (*Pan troglodytes*), females have been observed acting as mediators to reconcile individuals (generally males) during aggressive interactions [10–12], while in humans (*Homo sapiens*) the presence of grandmothers enhances grandchildren’s survival by increasing their foraging efforts [13–16].

Collectively, these studies have highlighted the importance of older females for their social group, and there is evidence that their loss can have dramatic effects on the rest of the group. In elephants, the death of matriarchs results in family unit splitting [17, 18] and increases the likelihood of calf death [19]. In ring-tailed lemurs (*Lemur catta*), the loss of the troop’s matriarch led to the eviction of distantly related kin [20], while in captive rhesus macaques (*Macaca mulatta*), matrilines with matriarchs received fewer wounds than matrilines without matriarchs [21]. However, since in many species matriarchs are often also alpha females, it is unclear whether the disruptive consequences of their loss on the family socio-dynamics are due to their influence as experienced females or rather to the important role of alpha females in the group. This is because high-ranking individuals commonly stabilize the social group through their interventions in ongoing conflicts in the attempt to appease opponents [5, 10, 22, 23]. Accordingly, the loss of dominant individuals has been found to increase social instability [11, 24, 25], to change social behaviors in the group [11, 26–28] and to affect individuals’ stress levels [29]. However, a detailed analysis of the behavioral and physiological consequences of the death or removal of non-alpha matriarchs is currently lacking.

Rhesus macaques are good models for studying the consequences of the removal of non-alpha matriarchs. They form large multi-male multi-female groups with an average group size between 10 and 80 individuals [30–32]. Male macaques generally emigrate to neighboring communities around sexual maturity at 4–5 years of age [33], while females remain in the natal group and form linear dominance hierarchies on the basis of their matrilineal kinship. Thus, matriarchs with large families are likely to both have and provide substantial social support (as mothers often aid their offspring in conflicts [34]) as well as to influence social dynamics within their matriline.

**Fig 1:**
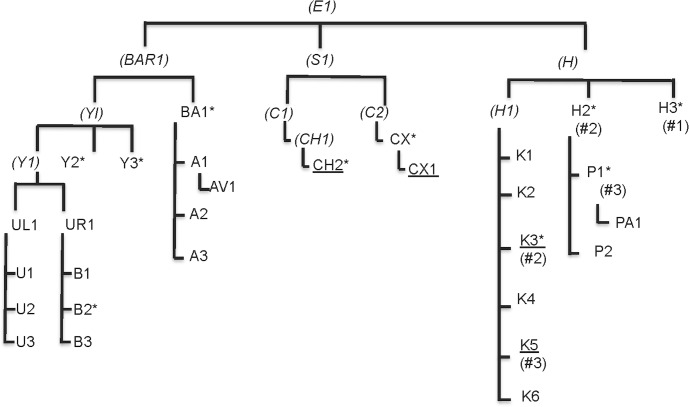
Pedigree of Matriline 3 members from a larger troop of rhesus macaques at the LCE field station. Deceased relatives are denoted by parentheses and italicized font. Underlines indicate males. (#) represents rank within sex (limited to #1–3 to show H1’s relationships to top-ranking members; Alpha male is from matriline 1). *denotes individuals with behavioral data

Here, we use the definition of a matriarch as the oldest living member of a family (where family is defined as descended from a common female ancestor). As females in our troop have their first offspring around 3–5 years of age, a family’s matriarch has at least a grandmother status. Because rank in rhesus macaques is determined by birth rank (i.e. mother’s rank) and not age [35], a matriarch is not necessarily the alpha female. We tested the hypothesis that the removal of a non-alpha matriarch in a captive colony of rhesus macaques significantly changed behavior and physiology in her matriline but not within the other matrilines present in the troop. At the time of this study, this matriarch, H1, had seven offspring in the population, and had birthed a total of 13 offspring overall, making her the matriarch with the largest number of extant offspring in the troop. Two of H1’s sons were high-ranking males (2^nd^ and 3^rd^) in the troop, and her two younger sisters were the troop’s alpha and beta females (with the beta’s eldest daughter, P1, outranking H1, and P2 and PA1 beginning to ascend the hierarchy but not yet ranking above H1, see Fig 1). Thus, although H1 was not one of the top three ranking females in the troop, it is likely that she exerted a strong influence on matrilineal dynamics.

We tested this hypothesis by retrospectively examining dominance stability, vigilance, grooming, aggressive interactions, and hair cortisol concentrations (HCCs) within H1’s matriline, while also examining the other two matrilines within the troop and overall troop wounding, before and after her removal. We assessed HCC as a measure of chronic hypothalamic-pituitary- adrenocortical (HPA) activity. HCC measurement has several advantages over the collection of other biological samples (i.e. blood, urine, and feces), including the ability to measure chronic HPA activity without the need of multiple sampling and without the confounds of circadian and environmental variability [36–38]. We hypothesized that the matriarch provided stability within the matriline, and predicted that her removal would lead to 1) rank changes, and thus higher instability (*prediction 1*), 2) higher aggression rates (*prediction 2*), and 3) higher levels of wounding (*prediction 3*). An increase of rank instability and aggression rates following the matriarch’s removal should, in turn, increase individuals’ vigilant behaviors (*prediction 4*) and should increase HCCs as an index of chronic stress, especially among high-ranking animals (*prediction 5)* as their dominance positions are contested [29]. Finally, previous work has shown how social grooming can decrease social tension among primates [39, 40], and thus we predicted rates of social grooming would increase during the period of social instability (*prediction 6*). Finally, if social grooming can help alleviate chronic stress, we also predicted that monkeys with the highest frequency of grooming would have relatively lower hair cortisol concentrations (*prediction 7)*. Because the matriarch is likely the most influential within her matriline, we did not predict any significant changes within the other two matrilines, which were presumably unaffected.

## Materials and Methods

### Subjects and housing

Subjects were 49 rhesus macaques (age range 3–17 years) born and reared at the Laboratory of Comparative Ethology (LCE) field station at the NIH Animal Center in Poolesville, Maryland, USA. Because there is an age-related decline in HCC in infants and juveniles that later stabilizes [41], we included all subjects above three years old. The field station is a 2.0 hectare outdoor open air enclosure consisting of natural vegetation, climbing structures, corncrib shelters, and a central pond measuring 0.09 hectares [42]. Continuous access to three indoor enclosures (2.74 x 5.79 x 4.27m) was also available, where animals had *ad libitum* access to water, as well as commercial lab diet (Purina Monkey Chow #5038, St. Louis, MO) and were given fresh fruits and vegetables twice daily. All procedures described below adhered to the NIH Guide for the Care and Use of Laboratory Animals and were approved by the NICHD Animal Care and Use Committee (ACUC). Furthermore, all applicable international, national, and/or institutional guidelines for the care and use of animals were followed.

### Group structure

The troop consisted of three major matrilines, which are related individuals descending from a female line. Matriline 3 has occupied the top rank since 2009, when its major families overthrew the previously dominant matriline 1 [42] leading to severe injuries and therefore the permanent removal of most members of matriline 1. Matrilineal hierarchies were ranked in the order, from highest to lowest: matriline 3, matriline 4, and matriline 1 (matriline 2 was removed in 2004 for management reasons) [42]. The demographic composition of the troop is shown in Table 1. Subjects were 49 adults (matriline 3: 29; matriline 4: 15; matriline 1: 5).

**Table 1:** Troop composition at the LCE field station in 2014.

	Adults (3+)	Juveniles/infants (<2)	Total
Matriline 3	29 (38%)	14 (18%)	43 (57%)
Matriline 4	15 (20%)	11 (15%)	26 (34%)
Matriline 1	5 (7%)	2 (3%)	7 (9%)
Total	49 (64%)	27 (36%)	76

Numbers in parentheses represent proportion out of entire troop (%).

### Demographic change: Removal of matriline 3 matriarch

In late September 2014, the troop’s eldest (but non-alpha) matriarch, H1 (18yrs), was removed from the field station due to chronic gastrointestinal issues that resulted in chronic diarrhea and weight loss and necessitated treatment that could not be provided in the field.

H1’s offspring remained in the troop after her removal, including six adults (five females, one male) and one juvenile female. However, the two youngest females were permanently removed from the population at the bi-annual health exam in February 2015 as part of routine population management.

### Data collection and analysis

#### Behavioral data

Behavioral data were collected using modified frequency data sheets [43] from eight females and two males (see Fig 1), representing every major lineage within matriline 3, through continuous 5-minute focal animal interval sampling [44]. Data were also collected from the three highest-ranking females from matriline 4 and the two highest-ranking adults in matriline 1 (the troop’s alpha male and one female). Due to limited researcher availability, focal behavioral data were only collected on this subset of the population.

The 5-min samples were divided into 20, 15-second intervals. For each interval, every behavior that occurred within the 15 seconds was recorded. The frequency of intervals in which the behaviors occurred was then calculated. The maximum number of intervals an animal could perform a behavior therefore in one session was 20 intervals. Focal subjects received one weekly AM session (9:00–12:00) and one weekly PM session (12:00–17:00) for ten consecutive weeks from August to December 2014 by three observers (inter-rater reliability >85%). Data used were part of a long-term data collection procedure for unrelated projects, and therefore, coders were blind to the hypotheses of the study.

**Fig 2:**
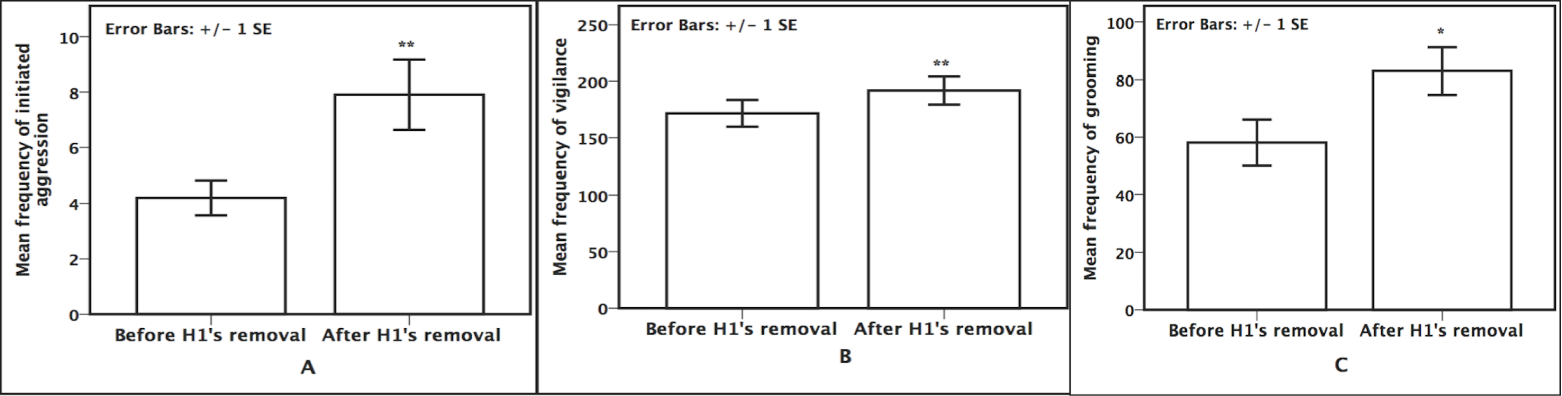
Frequencies of behaviors within matriline 3 before and after H1’s removal. Frequencies represent the average number of intervals (out of 400) the behavior occurred for each focal subject. **A. Aggression frequency** **p = 0.01. **B. Grooming frequency** **p = 0.01. **C. Vigilance frequency** *p = 0.02.

The focal animals within matriline 3 included: 1) the top three ranking females (H1’s sisters and niece: H3, H2, P1; see Fig 1); 2) a female who underwent a major rank change and rose above the matriarch’s offspring (CX); 3) a matriarch ranking immediately below H1’s family (BA1); 4) one son of the matriarch (2^nd^ in the male hierarchy, K3), 5) one high-ranking male (CH2, distant cousin to H1), 6) and three intermediate ranking females within the matriline (B2, Y2, Y3). From the focal animals, we recorded data on both social (e.g. aggression, social grooming) and non-social (e.g. feeding, vigilance) behaviors (only behaviors in Table 2 were analyzed for this study). In order to calculate rank changes and dominance stability, we noted all instances of dominance interactions (i.e. supplant, threat, chase, attack, and submissive) within the entire troop through *ad libitum* observations [44]. We collected 600 focal observations (40 sessions/focal animal, 20 before and 20 after), for a total of 50 hours of observations (25 hours before and 25 hours after H1’s removal), as well as 1,258 *ad libitum* troop dominance interactions [44].

**Table 2:** Behaviors and definitions collected during focal observations.

Behavior	Definition
Initiation of aggression	Displacements (taking the place of another), threats (open mouth, ears back, direct eye contact, includes lunges), attack (pin down and bite)
Grooming	Move hair apart and visual inspection, removal of debris; combination of given and received bouts
Vigilance	Scan the environment visually, at least 3 seconds

Behavioral observations thus included the ten focal subjects from matriline 3 and the five focal subjects from matrilines 4 and 1, while *ad libitum* aggressive interactions included all 49 subjects from across the three matrilines.

#### Dominance rank and social instability

Dominance hierarchies were calculated via Elo-rating, a numerical system that tracks rank changes over time by constantly updating values based on wins and losses [45–49]. We generated Elo-ratings using the *elo*.*sequence* function provided by Neumann et al. [45] in R software (v 3.1.2), by setting animals’ initial rating at 1,000, and the k factor, which is weighted based on the probability of winning, at 200. Two main advantages of the use of Elo-rating over conventional matrix-based analyses include the ability to track rank changes over time, and accommodating variations in social dynamics due to a fluctuations in the study population [45, 47], making Elo-rating ideal for this study.

Social instability was calculated through the *stability*.*index* function derived from Neumann et al. [45]. This function measures the ratio of rank changes per individuals present over a given time period based on the derived Elo-ratings. As Elo-ratings can be arranged to reflect ordinal ranks, fluctuations in Elo-ratings reflect higher levels of instability. According to Neumann et al. [45], S values typically range from 0 to 0.5, where higher S values reflect higher levels of instability, and lower values reflect a relatively stable hierarchy. Stability measures were taken for the three months before and after H1’s removal for each matriline. Dominance ranks and stability measures thus included data from all 49 subjects across the three matrilines.

#### Severe aggression

*Ad libitum* records of severe aggression (defined as interactions resulting in tissue damage) were routinely recorded as part of the long-term data collection procedures for this colony of animals. For this study, instances of severe aggression from August to December 2014 were examined. Because these types of social interactions are rare, *ad libitum* sampling is the optimal recording method. Severe aggression was analyzed to determine whether H1’s removal resulted in an increase in this behavior for her matriline compared to matrilines 4 and 1.

#### Hair sample collection and cortisol assay

Hair samples were collected by shaving the back of the animals’ necks [36] during routine health exams in August 2014 and February 2015 for all 49 subjects across the three matrilines. Because these animals had been shaved as part of a longitudinal study every six months beginning in Fall 2012, the samples in August 2014 reflected hair cortisol concentrations (HCCs) that had accumulated since February 2014, and the February 2015 samples reflected HCCs that had accumulated since August 2014 (thus reflecting the timing of H1’s removal in September 2014). Samples were stored in a foil pouch at -80°C until shipment to the Hormone Assay Core Laboratory at the University of Massachusetts Amherst. Following Dettmer et al. [41], samples were weighed, washed twice with isopropanol and dried for 5–7 days under a fume hood. Samples were then ground to a fine powder with a ball mill grinder (MM200; Retsch, Newtown, PA) and incubated in methanol for 24 hours to extract cortisol from the samples. Aliquots of the methanol extract were dried down and reconstituted with assay buffer, then analyzed via enzyme immunoassay (EIA) using a salivary cortisol kit (#1–3002; Salimetrics, State College, PA). Resulting values (ug/dL) were converted to pg/mg for analysis. Inter- and intra-assay coefficients of variation were <8% based on aliquots of the same extracted pooled hair sample analyzed repeatedly across assays.

#### Data analysis

Due to the small sample size of matriline 1 (N = 5), data for matrilines 4 and 1 were analyzed together for all analyses.

To assess whether focal animals changed frequencies in their behaviors following H1’s removal, we compared rates of each behavior collected approximately three months before and after her removal using paired-sample t-tests for both matriline 3 and the combined matrilines 4 and 1.

Subjects were classified as high or low ranking (within their respective matriline) based on a median split of the Elo-ratings. Levels of intense aggression (i.e. chases and attacks) were compared using both focal and *ad libitum* data using paired-sample t tests to examine whether high ranking monkeys increased in intense aggression.

Severe aggression was analyzed as the proportion of events that occurred across each matriline, (i.e. out of all recorded events, how many were from each matriline) and Fisher’s exact tests were used to compare changes in proportion of events for each matriline before and after H1’s removal.

HCC values were log transformed to ensure normality prior to analysis. Seven subjects were missing a HCC measurement in August (three from matriline 3, three from matriline 4, one from matriline 1), as they could not be captured during routine exams. Two subjects were also missing a HCC measurement in February (both from matriline 4; one of which did not have any HCC data for either time point). One subject from matriline 3 was also excluded from the August HCC analysis (as well as the HCC change analysis), as the HCC measurement in August was well above six standard deviations from the mean. We tested the association between individuals’ HCCs and their Elo-rating using Pearson’s correlation test for both matriline 3 and matrilines 4/1. Additionally, we ran Spearman’s correlation test (owing to the smaller sample sizes) between HCC changes (from August to February, in pg/mg) and Elo-rating changes for high and low ranking monkeys. We predicted that an increase in rank would be associated with an increase in physiological stress among high-ranking monkeys in matriline 3 as their dominance positions were contested.

To examine whether frequencies of social grooming were associated with HCCs after the matriarch’s removal, we calculated a grooming frequency across the entire 6 months (from August to February; the total amount of time that HCC accumulated) for the 15 subjects of whom behavioral data were collected (ten from matriline 3, five from matrilines 4 and 1). We then tested the association between grooming frequency and February 2015 HCC (which reflected chronic activity since August) using Spearman’s correlation test.

All tests were two tailed with the significance level set at p<0.05. SPSS 22 was used for analyses.

## Results

### Social stability and behavioral changes

As expected (*prediction 1*), we found lower social stability after H1’s removal compared to the three months before: the stability index for matriline 3 increased from 0.039 to 0.128. Matrilines 4&1 had little change in stability (from .017 to .018). Furthermore, our focal observations revealed that in the months immediately following H1’s removal, there was a significant increase in the initiation of aggression (paired-sample t-test: *t*_*(*9)_ = -3.20, *P* = 0.01, Fig 2A), in the time spent in vigilance (t_(9)_ = -3.15, *P* = 0.01, Fig 2B), and in grooming time *(t*_(9)_) *= -*2.74, *P* = 0.02, Fig 2C) within matriline 3, supporting *predictions 2*, *4* and *6*. No significant changes were found for the initiation of aggression *(t*_(4)_ *= 0*.10, *P* = 0.93), vigilance (*t*_(4)_ = -1.68, *P* = 0.17), or grooming (*t*_*(4)*_ = -1.33, *P* = 0.25) for matrilines 4 and 1.

The *ad libitum* and focal data revealed that high-ranking monkeys from matriline 3 also exhibited an increase in the levels of intense aggression (i.e., chases and attacks; mean ± SE: 0.67 ± 0.33 before to 7.60 ± 1.81 after; *t*_*(*14)_ = -3.86, *P* = 0.002) whereas low ranking animals did not show a significant change (*t*_*(*13)_ = -1.629, *P* = 0.13). There was no significant change in intense aggression for high ranking monkeys (*t*_(9)_ = -1.65, *P* = 0.13) or low ranking monkeys (*t*_(9)_ = -2.25, *P* = 0.05) within matrilines 4 and 1.

### Severe aggression

Contrary to our expectation (*prediction 3*), there was a reduction in overall troop severe aggression (from 9 to 6) compared to the period prior to H1's removal. However, when compared to the number of aggressive events experienced by the other matrilines, matriline 3 suffered proportionally more wounds after H1's removal. In the three months after her removal, no severe aggression was recorded for matrilines 4 and 1, while all recorded events occurred within matriline 3 (6 events total; *P*=0.04). In the three months prior to her removal, the matrilines had approximately equal numbers of severe aggression (four within matriline 3 and five within matriline 4 monkeys).

### Rank changes and hair cortisol concentrations

**Fig 3:**
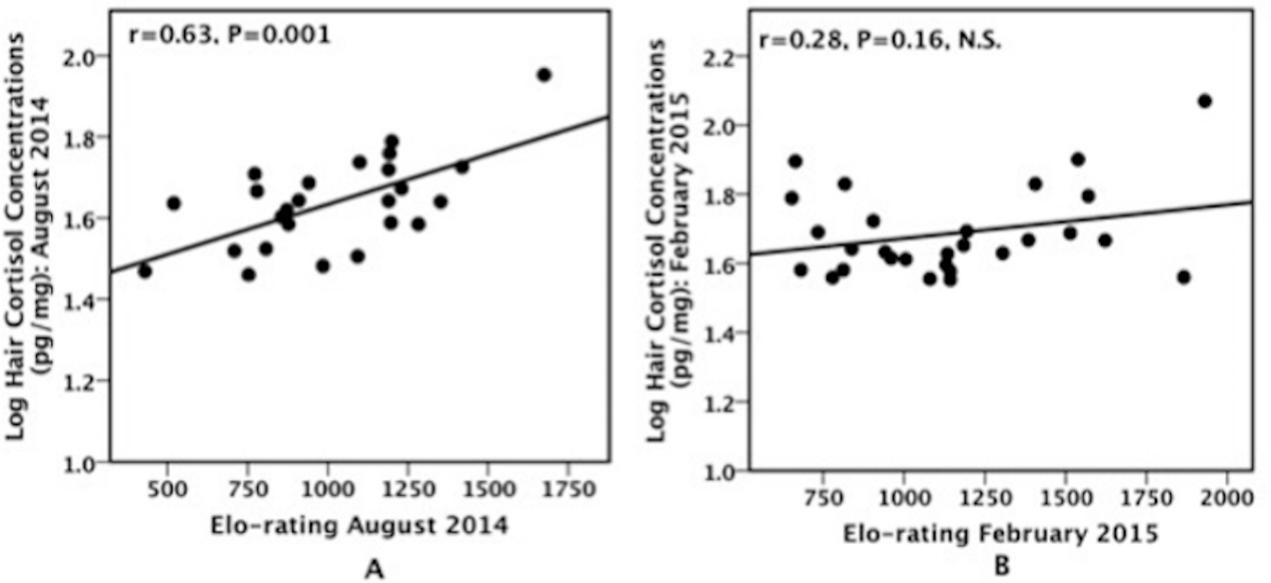
Relationship between rank and HCCs within matriline 3 before (3a:left panel) and after (3b:right panel) H1’s removal.

There was no significant change in HCCs from August 2014 to February 2015 for either matriline 3 (*t*_*(24)*_ = -1.05, *P* = 0.31) or matrilines 4 and 1 (t_(13)_ = 0.15, *P* = 0.89). For matriline 3, there was a significant positive correlation between Elo-rating and HCCs prior to H1’s removal in August (Pearson correlation: r = 0.63, *N =* 25, *P* = 0.001; see Fig 3A), but there was no correlation after her removal (r = 0.27, *N =* 28, *P* = 0.16, see Fig 3B). No significant correlations between Elo-rating and HCC were evident for matrilines 4 and 1 for either August (Pearson correlation: r = -0.23, N = 16, *P* = 0.39) or February (Pearson correlation, r = -0.42, N = 18, *P* = 0.08).

**Fig 4:**
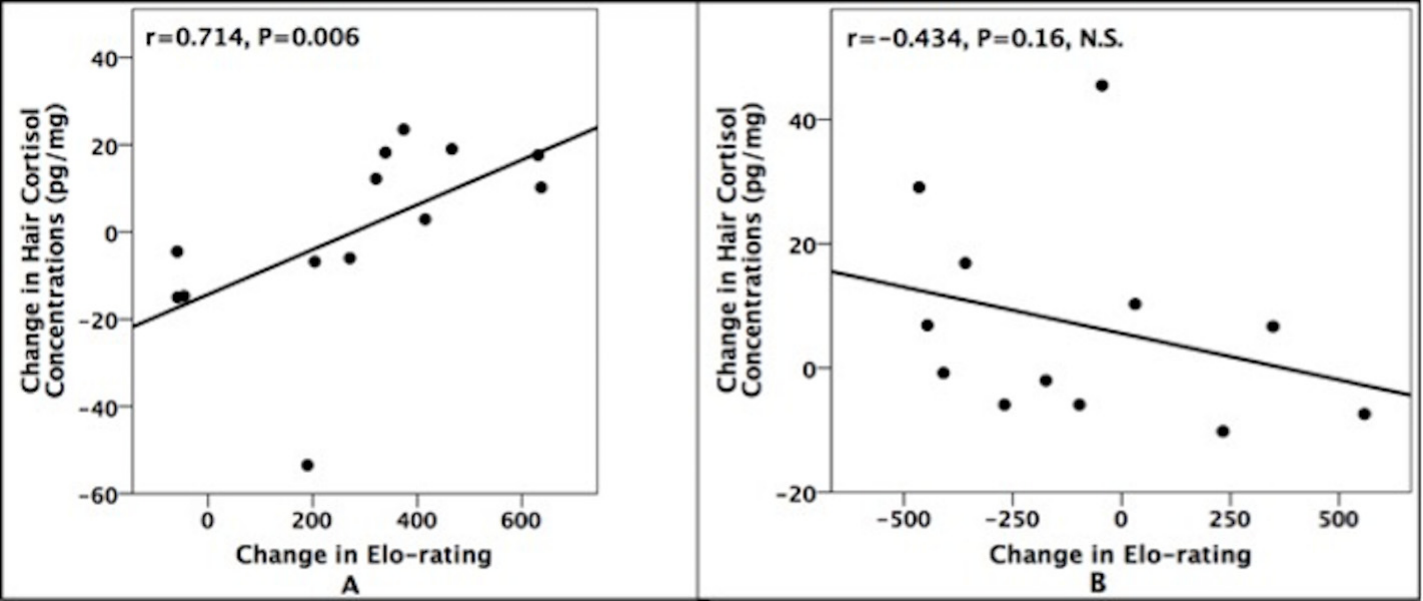
Relationship between rank change and HCC change in high-ranking (4a: left panel) and low-ranking (4b: right panel) rhesus macaques in matriline 3. Rank and HCC changes reflect changes from August 2014 to February 2015.

When comparing individuals based on high or low ordinal rank in matriline 3, the change in Elo-rating was positively correlated with the change in HCC (from August to February; Spearman, r_s_ = 0.71, *N =* 13, *P*<0.01, Fig 4A) for high-ranking monkeys, indicating that as top animals increased in dominance rank (and thus dominance assertion/increase in intense aggression), they experienced an increase in physiological stress, supporting *prediction 5*. There was no significant correlation for low-ranking animals (Spearman, r_s_ = -0.434 *N =* 12, *P* = 0.16, Fig 4B) within matriline 3. No relationship between Elo-rating change and HCC change was found for matrilines 4 and 1 for either high-ranking monkeys (Spearman, rs = -0.32, N = 7, *P* = 0.48) or low-ranking monkeys (Spearman, rs = -0.04, N = 8, *P* = 0.93; see S1 Dataset for data used in this current study).

### Hair cortisol concentrations and grooming frequency

**Fig 5:**
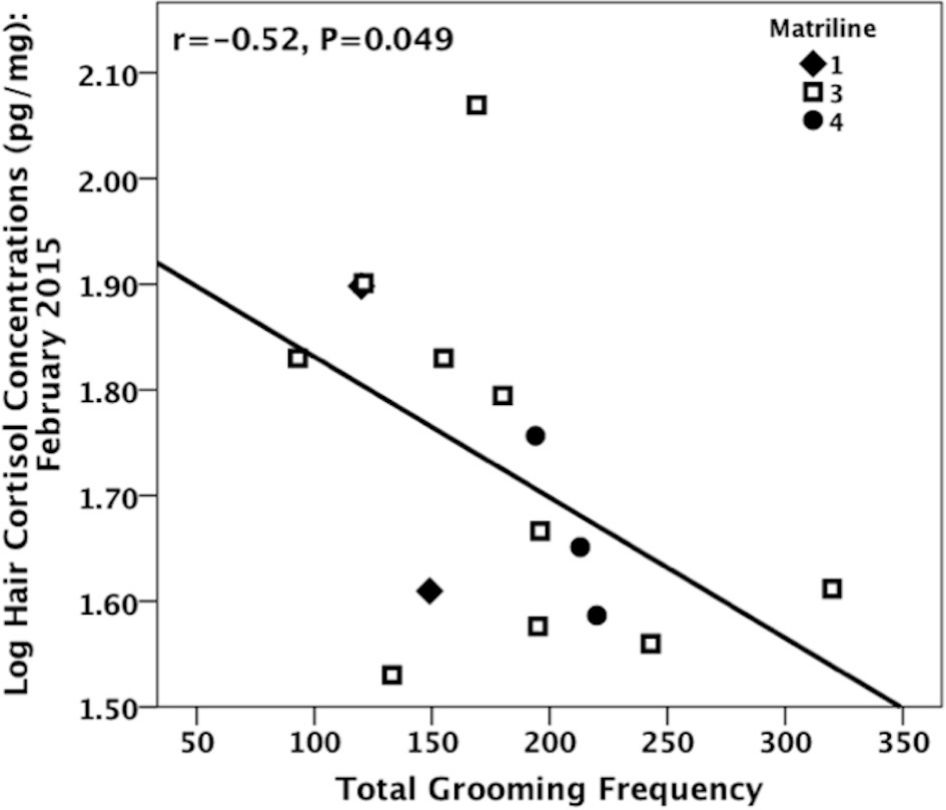
Total grooming frequency and HCC following H1’s removal. Frequency represents total number of intervals grooming occurred from August to February (when the HCC samples were taken).

While the total grooming frequency was not significantly associated with February HCC for matriline 3 (Spearman, r_s_ = -0.56, N = 10, P = 0.15, failing to support *prediction 7*), a significant negative association was evident when analyzing all subjects from which grooming data were collected (including the other two matrilines; Spearman, r_s_ = -0.52, N = 15, P = .049; see Fig 5).

## Discussion

Our data indicate that a non-alpha matriarch exerted a strong influence on her matriline: with direct ties to the dominant females and a large set of kin, her social ties were significant enough to influence dominance stability within her matriline, although she was not the alpha. Accordingly, in the period following H1’s removal, her matriline endured a period of social instability, with increased aggression and higher rates of both vigilance and social grooming. Interestingly, we also found that large increases in Elo-rating after H1’s removal were associated with large increases in HCC. These findings suggest that individuals within her matriline may have experienced higher levels of chronic stress, probably because in a period of frequent rank changes, each individual struggled to exert dominance over others (hence the increase of chase and physical attacks). The lack of any significant relation between rank changes and hair cortisol for the other matrilines suggests that only H1’s matriline was affected by her removal. We found that her matriline experienced both behavioral and physiological changes that resemble the consequences of the loss or takeover of alpha individuals described in both this [25] and other mammalian species (e.g. chacma baboons, *Papio ursinus* [26, 50] naked mole-rat, *Heterocephalus glaber* [24]; chimpanzees, *Pan troglodytes* [11]).

Our results are consistent with findings reported from a variety of species (chacma baboons, *Papio ursinus* [51–54]; wild dogs, *Lycaon pictus* [55]; long-tailed macaques, *Macaca fascicularis* [56]; African cichlid fish, *Haplochromis burtoni* [57]) showing that social stress in dominant individuals can be linked to social instability and the use of intense aggression by dominants to affirm their position. Interestingly, we found a positive association between dominance rank and hair cortisol before H1’s removal, suggesting that high-ranking individuals may experience more chronic physiological stress than subordinates under stable conditions. This result is at first puzzling since in the period preceding the matriarch’s removal, we found stable rank relationships within the matriline, and social stability is commonly associated with higher stress levels in *subordinates*, who receive continuous harassment from dominants (chacma baboons [29]; but see [58] for a discussion of moderating factors that influence the relationship between social rank and physiological stress in nonhuman primates). However, this relationship might be explained by the relatively new, but stable, hierarchy present in the troop (circa 1 year) due to a social overthrow within the matriline in late December 2013/early January 2014 [47]. It is possible therefore that there might have been some degree of social tension within the matriline even in the period *before* H1’s removal, that her presence might have helped to hold in check, through, for instance, policing interventions [22]. Consequently, after the removal of the matriarch, and the threat to this relatively new hierarchy, this social tension was no longer under control, leading to higher levels of social instability and stress. Unfortunately, we do not have data on policing behavior to test this hypothesis directly in this study. However, we do know that H1 managed conflict among her offspring and following her removal, there was a surge in offspring conflicts, as well as challenges to their dominance positions (e.g. CX).

Notably, we did not find any association between dominance rank and HCC in the months following H1’s removal, probably because only a restricted number of individuals experienced heightened levels of stress during social instability, namely those high-ranking monkeys who increased in Elo-rating and intense aggression. Furthermore, since grooming has been shown to decrease animal stress levels [59–62] it is plausible that the increase in social grooming we found in H1’s matriline after her removal might have been a strategy monkeys used to counter-act the increased instability, and this, in turn, minimized the surge in social and physiological stress in this period. In wild chacma baboons, females who experienced increased stress levels following the removal of close relatives displayed higher rates of grooming which likely helped them to return cortisol concentrations to baseline levels [63]. While we did not find a significant relationship between total grooming frequency and HCCs specifically for H1’s matriline, we did find a significant negative relationship when analyzing all subjects for whom we had behavioral data recorded, indicating that social grooming may be an important mechanism to reduce chronic physiological stress. It is possible that with more subjects from her matriline, we would have found a significant relationship between hair cortisol and grooming specifically for her matriline. Importantly, our work is one of the few to relate social rank to HCCs in primates [64,65], and mammals in general, although one study found a positive correlation between rank and HCC in hyraxes (*Procavis capensis* [66]). Our study suggests that HCCs may be useful in assessing how social dynamics influence chronic stress in group-living mammals.

Following H1’s removal, instances of severe aggression were only recorded for her matriline, indicating that high intensity aggression was more frequent within her matriline. These findings are underscored by our observation of increased chases and attacks by the high-ranking moneys of matriline 3 after H1’s removal. The increase in dominance instability, and severe physical aggression, were the likely consequences of monkeys trying to reaffirm their rank. In support of this, one month after the matriarch’s removal, CX had undergone a major rank increase and ranked above all seven of H1’s offspring. During this time, she was also observed with a large tear wound. This wound was likely a byproduct of CX attempting to increase in rank (successfully), as well as the unwillingness of the others to sacrifice their positions (those attempting to reaffirm their position). Unfortunately, wounds are rarely ever seen occurring, so it is impossible to know exactly who inflicted this wound. The fact that no severe aggression was observed within the other matrilines suggests that they were likely not struggling to assert dominance over one another, a notion supported by their relatively stable hierarchies. Furthermore, the increased levels of intense aggression including those resulting in tissue damage may have resulted from changes in submissive signaling. Submissive signals (such as the silent bared teeth display) have been related to lower levels of severe aggression [67] and are associated with greater dominance relationship certainty [68]. While submissive displays were recorded, they were originally not a focus of *ad libitum* sampling and occurred too infrequently during focal observations, and therefore direct comparisons were not available during this study. Therefore, an interesting opportunity exists in the future to study the role and changes in submissive signaling and resultant levels of aggression during a time of social instability such as after the loss of a matriarch.

As a likely result of the increased levels of aggression and observed tissue damage, there was also an increase in the frequency of vigilance. In a time of rank changes and monkeys attempting to reaffirm their rank, vigilance may have been a buffering mechanism from both directed and redirected aggression. Additionally, monkeys may have increased in vigilance to monitor the social interactions of others. Indeed, female mountain gorillas (*Gorilla gorilla beringei*) spend more time monitoring individuals whom they had aggressive interactions [69] and may play a role in conflict-avoidance in tufted capuchins (*Cebus apella*; [70]).

Importantly, it is worth noting that the matriarch’s removal happened in the midst of the breeding season and thus the variation in aggression rates and possibly wounding rates we found in our study might be related to increased competition during the breeding season [71, 72]. However, we did not find any evidence for changes in observed severe aggression in the previous 2013 breeding season: with matrilines 3 and 4 each observed with approximately five instances of tissue damage in each time period identical to this study. In addition, we found a reduction in the overall number of severe aggression resulting in tissue damage in 2014 (from before the start of the breeding season to after), indicating that high levels of aggression may not be limited to the breeding season. Furthermore, because our findings (grooming, vigilance, aggression, HCC, rank, instability, etc.) were only significant for the matriarch’s matriline (matriline 3), and not the other matrilines (matrilines 4 and 1), it is unlikely that the findings are solely a result of the breeding season alone. However, data from matrilines 4 and 1 should be taken with caution due to the small sample sizes.

More generally, our results emphasize the importance of careful consideration in the management of a captive colony when deciding whether some individuals should be permanently removed. Although each individual primate colony is unique and management decisions will differ across facilities, it is important for researchers, colony managers, and veterinarians to consider that group members who play a key role for the stability of the social group should be identified, even if they are not among the alpha individuals. Our study shows how the removal of even non-alpha matriarchs significantly impacts matrilineal dynamics: their absence can trigger a period of social instability and heightened intense aggression. In the case in which socially significant individuals must be removed, extra monitoring is recommended to detecting potential changes in stability. Managers of captive colonies may therefore be able to use data generated via Elo-rating and rank changes (i.e. social instability) to examine how certain characteristics (age, rank, number of kin, rate of policing behavior, etc.) of an individual may be important in maintaining stability. This will then in turn allow managers to predict the effects of the removal of certain individuals in the future and guide future management decisions.

In conclusion, this study provides evidence that old-aged matriarchs may play a substantial role in family stability in group-living primates. When considering the stability of large family groups, it may be important to recognize the critical role that non-alpha matriarchs provide. Collectively, our findings demonstrate the power of specific individuals, including non-alpha group members, to exert strong influences within their group and the consequences following their loss.

## Supporting Information

S1 Dataset Dataset for the current study in excel format (XLSX)
