# Correction: Matrilineal Behavioral and Physiological Changes following the Removal of a Non-Alpha Matriarch in Rhesus Macaques (*Macaca mulatta*)

**DOI:** 10.1371/journal.pone.0167739

**Published:** 2016-11-30

**Authors:** Lauren J. Wooddell, Stefano S. K. Kaburu, Kendra L. Rosenberg, Jerrold S. Meyer, Stephen J. Suomi, Amanda M. Dettmer

## Notice of Republication

An incorrect version of this paper was published. The article was republished on November 11, 2016 to provide the correct version. Please download this article again to view the corrected PDF.

## References

[1] Wooddell LJ, Kaburu SSK, Rosenberg KL, Meyer JS, Suomi SJ, Dettmer AM (2016) Matrilineal Behavioral and Physiological Changes following the Removal of a Non-Alpha Matriarch in Rhesus Macaques (*Macaca mulatta*). PLoS ONE 11(6): e0157108 doi:10.1371/journal.pone.0157108 27275743 10.1371/journal.pone.0157108 PMC4898773

